# The effect of web‐designed education on medication adherence, asthma control and fatigue in patients with asthma: A randomized controlled trial

**DOI:** 10.1111/ijn.13288

**Published:** 2024-07-19

**Authors:** Eylül Gülnur Erdoğan, Özlem Örsal

**Affiliations:** ^1^ Department of Public Health Nursing, Faculty of Health Science Bilecik Seyh Edebali University Bilecik Turkey; ^2^ Department of Public Health Nursing, Faculty of Health Science Eskisehir Osmangazi University Eskisehir Turkey

**Keywords:** asthma, asthma control, fatigue, medication adherence, web‐designed education

## Abstract

**Aims:**

This study aimed to determine the effect of web‐designed education developed for asthma patients on drug adherence, asthma control and fatigue.

**Methods:**

This randomized controlled trial was conducted between August 2021 and January 2022 with 200 individuals suffering from poor asthma control who participated in web‐designed education. After the intervention, the asthma patients were followed up for 6 weeks to measure Medication Adherence Report Scale (MARS), Chronic Obstructive Pulmonary Disease and Asthma Fatigue Scale (CAFS), Asthma Control Test (ACT) and Inhalation Devices Usage Techniques Knowledge Test (IDUSTKT). Data were analysed in the Statistical Package for the Social Sciences program using the Chi‐square test, Independent *t*‐test, Man–Whitney *U* test, Wilcoxon test, Paired *t*‐test, Greenhouse–Geisser (*F*) test and Linear regression.

**Results:**

The web‐designed education had a statistically significant effect on the total scores of CAFS, ACT and IDUSTKT for individuals with asthma (*p* < 0.001). This intervention decreased fatigue levels, improved asthma control and enhanced knowledge of inhalation device usage techniques. Although there was an improvement in medication adherence, this difference was not statistically significant.

**Conclusion:**

These results suggest that web‐based educational programs can be an effective tool in asthma management and may improve patients' quality of life. Future research should examine the long‐term effects of such educational programs and their effectiveness across different demographic groups in more detail.

## Summary statement

What is already known about this topic?

- Asthma is a common public health problem, and it affects individuals' physical and social lives.
- There are some studies in the literature on the prevalence and treatment of asthma, but there are few studies on web‐based training and counselling programs.

What this paper adds?

This study demonstrates that web‐designed asthma education and counselling programs can improve asthma control, fatigue levels, inhalation device usage techniques knowledge and some accompanying problems in patients with asthma.

The implications of this paper:

- Health personnel and administrators should be aware of the potential benefits of web‐designed asthma education and can recommend the implementation of the program.
- A web‐designed approach helps to reach patients who do not want to participate in a traditional asthma education program.

## 1 INTRODUCTION

Asthma is a chronic inflammatory disease associated with airway hyperresponsiveness, characterized by coughing attacks, shortness of breath, recurrent wheezing and chest tightness, especially at night or early in the morning (Becker & Abrams, 2017). In addition to the main symptoms of the disease, individuals with asthma experience fatigue, lack of energy and daytime drowsiness (Rudell et al., 2012; Svedsater et al., 2017; Teodorescu et al., 2006). Fatigue is a common symptom in patients with chronic diseases. Although the proportion of people who feel tired in the general population reaches 20%, it is much higher in those with chronic lung disease and reaches up to 80% in those with restrictive lung disease (de Kleijn et al., 2009; Drent et al., 2012). Peters et al. (2014) found that fatigue was the most common cause of impaired quality of life among 167 patients with severe asthma (90.4%). Therefore, fatigue may be a common and clinically significant symptom in patients with asthma (Landmark‐Høyvik et al., 2010). There is a notable negative correlation between the severity of fatigue and the level of quality of life. Managing fatigue is crucial for enabling these patients to carry out daily activities and maintain a good quality of life (Kouijzer et al., 2018).

Inhalers prescribed to control asthma symptoms help to improve quality of life and reduce the risk of flare‐ups or exacerbations. However, there is evidence that many people with asthma do not use their inhalers correctly (Normansell et al., 2017). Patients receive inadequate drug doses due to inappropriate inhaler techniques. This limits the efficacy of the drug and may also cause side effects due to drug exposure on the back of the throat and mouth (Melani et al., 2011; Price et al., 2013). Although the patient takes the medication as prescribed, incorrect inhaler technique can lead to poor disease control. Consequently, patients may alter their medication dosages and resort to arbitrary medication changes, ultimately leading to emergency department visits or hospitalizations as a result of poor symptom control (Gibson et al., 2013; Price et al., 2013). Since inhaler use is considered easy, many patients and healthcare workers do not receive inhaler training or receive inadequate training. One study found that 39%–67% of nurses, physicians and respiratory therapists were unable to adequately identify or implement critical steps related to inhaler use (Fink & Rubin, 2005). Effective management of asthma requires a partnership between the individuals with asthma and healthcare providers, and this needs to be personalized for each patient (GINA, 2020; Taylor et al., 2018).

Web‐designed education, which is becoming increasingly widespread in the world and provides many advantages in patient education, is accepted as an effective method of providing health education (Altıntaş & Vural, 2018). Studies in the literature showed that web‐designed methods are used to improve asthma control, medication adherence problems and quality of life of individuals with asthma (Ahmed et al., 2016; Koufopoulos et al., 2016; Luyster et al., 2020). A major rationale for designing such a system for asthma is that respiratory health problems have become widespread in the world and threaten public health. Therefore, it is very important that individuals with asthma take their asthma medication correctly and regularly without putting themselves at risk. In light of this information, the present study aimed to investigate the effect of web‐designed education with text or call reminders and counselling interventions on medication adherence, asthma control and fatigue in patients with asthma.

## 2 METHODS

### 2.1 Study design and participants

**FIGURE 1:**
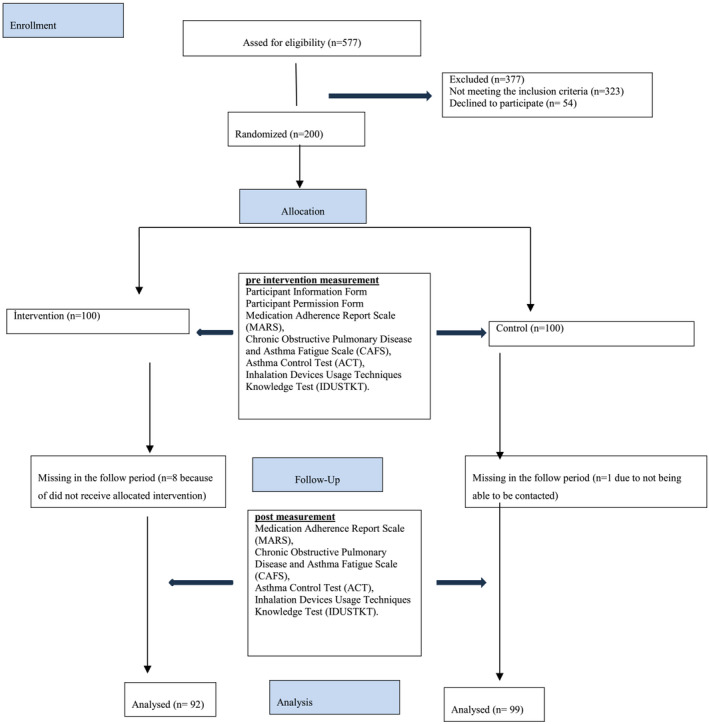
CONSORT flow diagram for the study.

This study utilized a two‐group, parallel randomized‐controlled single‐blind design. This randomized controlled trial was conducted with a study population consisting of patients diagnosed with asthma registered in family health centres in Eskişehir. The inclusion criteria for participants were that they must be at least 18 years old, have been diagnosed with asthma for at least 1 year, have poor asthma control and use at least one asthma medication. Participants who met the inclusion criteria and completed the pretest were randomly assigned to either the intervention or control groups using a computer‐generated random number sequence. A total of 577 participants responded to the eligibility survey, with 200 participants included in the study after completing the pretest. The experimental group (*n* = 100) received web‐based training, whereas the control group (*n* = 100) received conventional care without additional interventions. Eight patients in the experimental group did not complete the 6‐week follow‐up. The CONSORT flow diagram for the study is shown in Figure 1. Additionally, the study protocol has been registered at ClinicalTrials.gov (Trial Number: NCT04607681).

### 2.2 Hypotheses

We hypothesize that individuals with asthma who receive the web‐based asthma education program will demonstrate better asthma control, lower fatigue levels and higher medication adherence compared to those who do not receive the program.

### 2.3 Study group and power analysis

The G‐POWER 3.1 program was used to determine the sample size of the study. The effect size was calculated as (*d* = 0.5) based on the data from the study by Arslan and Öztunç (2013). Power analysis conducted to determine the sample size of the study (0.5 effect size, 95% power and 5% type 1 error) indicated a sample size of 92 in each of the experimental and control groups. Allowing for 10% attrition, we increased the sample size to 200 participants (100 participants per group) at baseline.

### 2.4 Randomization

Simple randomization was employed in this study, utilizing a computer‐generated sequence of random numbers for random assignment. An independent statistician conducted the group assignments, providing the researcher with a pre‐intervention list detailing individuals' assigned groups and respective intervention categories. This ensured both random assignment and blinded assignment, meeting the key requirements of randomization. Participants were unaware of when they would receive the web‐based education, ensuring participant blinding and preventing anticipation of group assignment, thereby fostering an objective educational experience. To minimize statistical and reporting biases, experts independent of the research team were consulted for data collection, outcome measurements, statistical analysis and reporting.

## 3 DATA COLLECTION TOOLS

Data collection was conducted from August 2021 to January 2022. The pretests before randomization were administered by the researcher immediately after obtaining written consent from individuals. To collect the study data, a researcher‐administered questionnaire for demographic data, in addition to the Asthma Control Test (ACT), Chronic Obstructive Pulmonary Disease and Asthma Fatigue Scale (CAFS), Medication Adherence Report Scale (MARS), and Inhalation Devices Usage Techniques Knowledge Test (IDUSTKT) evaluation form, was used. The questionnaires were administered online using a survey link created on Google Forms. Posttests were conducted after the 6th week of the study by researcher.

### 3.1 The questionnaire

The questionnaire was prepared by reviewing the literature (Bozbaş & Ulubay, 2011; Cingil et al., 2009; Kepil & Özgüçlü, 2017). The questionnaire included closed‐ended questions related to socio‐demographic characteristics (age and gender), general health status (presence of chronic diseases and asthma‐related conditions) and health behaviours (medication use and pet ownership).

### 3.2 Asthma Control Test (ACT)

The ACT was developed by Nathan et al. (2004), and its Turkish validity and reliability study was conducted by Uysal et al. (2013). The scale consists of 5 items in total, and each item is scored on a 5‐point Likert‐type scale (1 = ‘completely’, 2 = ‘mostly’, 3 = ‘occasionally’, 4 = ‘rarely’ and 5 = ‘never’). This test yields a maximum score of 25 and a minimum score of 5. A total score of 25 points is considered ‘full control’, 20–24 points as ‘good control’ and <19 points as ‘no control’ (Uysal et al., 2013). The Cronbach's Alpha coefficient was found to be .84. In this study, the Cronbach's Alpha coefficient for the ACT ranged from .83 to .93.

### 3.3 Chronic Obstructive Pulmonary Disease and Asthma Fatigue Scale (CAFS)

The CAFS was developed by Revicki et al. (2010), and its Turkish validity and reliability study was conducted by Arslan and Öztunç (2013). The scale consists of 12 items graded on a 5‐point Likert scale (1 = ‘never’, 2 = ‘rarely’, 3 = ‘sometimes’, 4 = ‘often’ and 5 = ‘very often’). Higher scores indicate higher levels of fatigue (Arslan & Öztunç, 2013). The Cronbach's Alpha coefficient was found to be .84. In this study, the Cronbach's Alpha coefficients for the ACT ranged from .75 to .88.

### 3.4 Medication Adherence Report Scale (MARS)

The MARS was developed by Horne and Hankins (2001) to assess medication adherence and can be customized according to disease type. A Turkish validity and reliability study was conducted by Şen et al. (2019). The scale consists of 5 items graded on a 5‐point Likert scale (1 = ‘always’, 2 = ‘frequently’, 3 = ‘sometimes’, 4 = ‘rarely’, 5 = ‘never’). The total score is calculated by summing the scores obtained from the questions. Higher total scores indicate better adherence, and lower scores indicate a lack of adherence. The Cronbach's Alpha coefficient was found to be .78. In this study, the Cronbach's Alpha coefficients for the ACT ranged from .93 to .94.

### 3.5 Inhalation Devices Usage Techniques Knowledge Test (IDUSTKT)

This form was developed by the researchers based on the training videos on the use of inhalation devices to evaluate the skills of individuals with asthma in using inhalation devices. The drugs used were categorized, and separate evaluation questions were created for each drug category. Patients were asked to fill out this form according to the category of medicines they were prescribed. Participants are asked to answer the statements in the form as ‘Yes’ or ‘No’, and 1 point is given for correct answers. The higher the score, the higher the level of knowledge regarding the use of inhalation device usage techniques. The form included 16 questions on the use of metered‐dose inhalers, 14 questions on the use of dry‐powder inhalers‐1 (inhalers used by placing the drug in capsule form into the cavity inside the device), 13 questions on the use of dry‐powder inhalers‐2 (inhalers with the drug to be applied in powder form) and nine questions on the use of nebulizers.

### 3.6 Procedure and intervention

In the first interview with patients included in the intervention group, the researcher administered the Individual Descriptive Characteristics Form, Information Requirement Determination Questionnaire, CAFS, MARS, ACT and IDUSTKT via Google Forms.

The usernames and passwords of the patients in the intervention group were created by the researcher. Steps to log in to the website were explained in a video, and the link was sent to the patients via WhatsApp. Topics covered in the asthma education program include asthma symptoms, risk factors, diagnosis and treatment processes, methods of protection from triggers, treatment systems, correct inhaler use techniques and alternative treatment options. Additionally, there are 10 pre‐recorded videos demonstrating the use of commonly prescribed asthma medications. The educational topics are sequentially presented and designed such that each topic must be completed before proceeding. Patients were asked to review all the topics on the website and to use the website at least once a week. During the research, participants in the intervention group were able to communicate and consult with the researchers regarding any problems they experienced while using the website. The researchers responded to the questions submitted by the patients within 24 h at the latest. The answers were sent both to the patients' accounts and to their cell phones as text messages. The page also contained an administration panel managed by the researchers. Usage statistics, the topics read by the patients and the videos watched were monitored via the administration panel. The daily use of the website by the participants in the intervention group was monitored by the researchers. Patients who did not use the website were encouraged to use the website by calling them on the phone or sending them a reminder message. Six weeks later, a posttest was applied, and the participants completed the CAFS, MARS, ACT and IDUSTKT online. Participants also filled in the evaluation form created by the researchers to review the website. The follow‐up period lasted a total of 6 weeks.

Participants in the control group were expected to continue their usual/routine activities for 6 weeks after baseline assessment and randomization. Control group participants were asked not to participate in any new or intervention training programs during the 6‐week study period. The control group was also assessed at baseline and after the 6th week. The researcher did not directly intervene in this group.

### 3.7 Ethical considerations

Ethical committee approval and institution permission to conduct the research was obtained before the study commenced (30/10/2020, E‐80558721‐050.99‐105748). Before starting the study, written informed consent was obtained from the individuals who agreed to participate in the study by reading the protocol information to them orally. Approval of the authors was obtained to use the scales used in the study. After the end of the research, the control group was also a program for 6 weeks in the research were provided to them.

### 3.8 Data analysis

The data were analysed using IBM SPSS V23 (Armonk, NY). We used descriptive statistics for the descriptive variables, specifically, number (*n*), percentage (%), mean (*X*), standard devitation (*SD*), and median, and compared categorical variables using the Chi‐square test. The conformity of the data to normal distribution was examined by the Shapiro–Wilk test. Student's *t*‐test was used for the comparison of normally distributed variables between two independent groups, and Paired *t*‐test was used for the comparison of pretest and posttest measurements. Mann–Whitney *U* test was used to compare non‐normally distributed variables between two independent groups. Intention‐to‐treat analysis (ITT), expectation maximization method and missing observation analysis were used for missing participants to maintain the effect of randomization and to prevent attrition bias. Linear regression analysis was performed to determine the effect of web‐based training on various outcome measures. For all statistics, we set *p* < 0.05 as the level of significance.

## 4 RESULTS

There were no statistically significant differences between the intervention and control groups in terms of descriptive features at the beginning of the study (*p* > 0.05; Table 1). There were 94 individuals (51.6%) in the intervention group and 89 individuals (50.6%) in the control group who used spray medication. Additionally, 17 individuals (9.3%) in the intervention group and 12 individuals (6.8%) in the control group used dry‐powder inhalers. The distribution of asthma‐related medication usage in the study groups was statistically similar (*p* > 0.05). Furthermore, there were 33 individuals (33%) in the intervention group and 29 individuals (29%) in the control group who used one medication. Moreover, 55 individuals (55%) in the intervention group and 66 individuals (66%) in the control group used two medications. The distribution of the number of asthma‐related medication uses in the study groups was statistically similar (*p* > 0.05; Table 2).

**TABLE 1:** Demographic characteristics of the study groups (*n* = 200).

Variables	Intervention		Control		Total		Test (*p*)
	*n*	%	*n*	%	*n*	%	
Age group							*χ* ^2^ = 4.417 *p =* 0.220
18–28 years	22	22.0	28	28.0	50	25.0	
29–39 years	27	27.0	32	32.0	59	29.5	
40–50 years	41	41.0	27	27.0	68	34.0	
≥51 years	10	10.0	13	13.0	23	11.5	
Gender							*χ* ^2^ = 0.893 *p =* 0.345
Female	75	37.5	69	69.0	144	72.0	
Male	25	12.5	31	31.0	56	28.0	
Marital status							*χ* ^2^ = 1.156 *p =* 0.282
Married	73	73.0	66	66.0	139	69.5	
Single	27	27.0	34	34.0	61	30.5	
Education status							*χ* ^2^ = 0.255 *p =* 0.968
Literate/Primary school	19	19.0	21	21.0	40	20.0	
Middle school	8	8.0	8	8.0	16	8.0	
High school	40	40.0	41	41.0	81	40.5	
University/Graduate	33	33.0	30	30.0	63	31.5	
Social security status							*χ* ^2^ = 1.000 *p =* 0.500
Yes	96	96.0	95	95.0	191	95.5	
No	4	4.0	5	5.0	9	4.5	
Employment status							*χ* ^2^ = 0.200 *p =* 0.887
Working	51	51.0	52	52.0	103	51.5	
Not working	49	49.0	48	48.0	97	48.5	
Family income status							*χ* ^2^ = 0.990 *p =* 0.952
Income > Expense	18	18.0	19	19.0	37	18.5	
Income = Expense	71	71.0	69	69.0	140	70.0	
Income < Expense	11	11.0	12	12.0	23	11.5	
Presence of a chronic disease other than asthma							*χ* ^2^ = 0.528 *p =* 0.467
Yes	41	41.0	36	36.0	77	38.5	
No	59	59.0	64	64.0	123	61.5	
Presence of other asthmatic members in the family							*χ* ^2^ = 3.720 *p =* 0.054
Yes	21	21.0	11	11.0	32	16.0	
No	79	79.0	89	89.0	168	84.0	
Smoking status							*χ* ^2^ = 0.221 *p =* 0.638
Yes	27	27.0	30	30.0	57	28.5	
No	73	73.0	70	70.0	143	71.5	
Pet ownership at home							*χ* ^2^ = 4.063 *p =* 0.440
Yes	36	36.0	23	23.0	59	29.5	
No	64	64.0	77	77.0	141	70.5	

*Note*: Chi‐square test (*χ* ^2^); descriptive statistics are given as numbers (*n*) and percentage (%) values.

**TABLE 2:** Drug use characteristics of the study groups (*n* = 200).

Variables	Intervention		Control		Total		Test (*p*)
	*n*	%	*n*	%	*n*	%	
Type of inhaler used^a^							*χ* ^2^ = 1.152 *p =* 0.765
Metered‐dose inhaler	94	51.6	89	50.6	183	51.1	
Dry‐powder inhaler‐1	17	9.3	12	6.8	29	8.1	
Dry‐powder inhaler‐2	63	34.6	65	36.9	128	35.8	
Nebulizer	8	4.4	10	5.7	18	5.0	
Number of drugs							*χ* ^2^ = 5.401 *p =* 0.145
1 piece	33	33.0	29	29.0	62	31.0	
2 piece	55	55.0	66	66.0	121	60.5	
3 piece	9	9.0	5	5.0	14	7.0	
4 piece	3	3.0	0	0.0	3	1.5	

*Note*: Chi‐square test (*χ* ^2^); descriptive statistics are given as number (*n*) and percentage (%) values.

^a^ Those who answered yes were evaluated.

At the measurement times, MARS averages did not show a statistically significant difference between the groups. In the intervention group, the mean MARS score in the posttest was statistically higher than that in the pretest. Conversely, in the control group, the mean MARS score in the posttest was statistically lower than that in the pretest. Consequently, there was a statistically significant increase of 1.14 ± 3.28 units in MARS values in the intervention group and a statistically significant decrease of −0.58 ± 2.15 units in the control group. CAFS averages at the time of the pretest measurement did not show a statistically significant difference between the groups. However, at the posttest measurement time, the mean CAFS score in the intervention group was statistically lower than that in the control group. In the intervention group, the mean CAFS score in the posttest was statistically lower than that in the pretest. In contrast, in the control group, there was no statistically significant difference observed between the pretest and posttest scores. Consequently, there was a statistically significant decrease of 14.83 ± 14.33 units in CAFS values in the intervention group, whereas the change of 1.73 ± 10.59 units in the control group was not statistically significant. Group main effect was found to be statistically significant for CAFS, ACT, metered‐dose inhaler, dry‐powder inhaler‐1 and dry‐powder inhaler‐2 scores (*p* < 0.05). Additionally, when examining the group effect in the group‐time interaction (group*time), it was observed that the effect of the group on the change in CAFS, ACT, metered‐dose inhaler, dry‐powder inhaler‐1 and dry‐powder inhaler‐2 was significant (*p* = 0.001; Table 3).

**TABLE 3:** Comparison of pretest and posttest scores of the intervention and control groups.

Variables		Intervention	Control	Test statistics^b^		Group and time effect
		*X* ± *SD*	*X* ± *SD*	*p*	*d* (GA 95.0%)	
MARS						Group; *F* = 0.002; *p* = 0.961 Time; *F* = 7.626; *p* = 0.158 Group*Time; *F* = 18.538; *p* = 0.091
Pretest		18.19 ± 5.00	19.00 ± 4.82	0.245	−0.17 (−0.44; 0.11)	
Posttest		19.32 ± 4.82	18.42 ± 4.49	0.188	0.19 (−0.09; 0.48)	
Test statistics^a^	*p*	**0.001**	**0.009**			
	*d* (GA 95.0%)	**−0.35 (−0.56; −0.14)**	**0.27 (0.07; 0.47)**			
Difference		1.14 ± 3.28	−0.58 ± 2.15	**<0.001**	**0.62 (0.33; 0.91)**	
CAFS						Group; *F* = 10.55; *p* < **0.010** Time; *F* = 52.182; *p* < **0.001** Group*Time; *F* = 83.269; *p* < **0.001**
Pretest		61.25 ± 18.22	60.83 ± 17.49	0.869	0.02 (−0.25; 0.30)	
Posttest		47.21 ± 15.31	62.85 ± 16.73	**<0.001**	**−0.97 (−1.27; −0.67)**	
Test statistics^a^	*p*	**<0.001**	0.108			
	*d* (GA 95.0%)	**1.04 (0.78; 1.29)**	−0.16 (−0.36; 0.04)			
Difference		−14.83 ± 14.33	1.73 ± 10.59	**<0.001**	**−1.32 (−1.63; −1.01)**	
ACT						Group; *F* = 75.753; *p* = **0.001** Time; *F* = 163.777; *p* = **0.001** Group*Time; *F* = 158.154; *p* = **0.001**
Pretest		10.90 ± 3.59	9.88 ± 3.20	**0.035**	**0.30 (0.02; 0.58)**	
Posttest		16.84 ± 4.08	9.85 ± 3.74	**<0.001**	**1.79 (1.45; 2.12)**	
Test statistics^a^	*p*	**<0.001**	0.84			
	*d* (GA 95.0%)	**−1.55 (−1.85; −1.24)**	−0.02 (−0.22; 0.18)			
Difference		5.78 ± 3.74	0.05 ± 2.48	**<0.001**	**1.82 (1.48; 2.16)**	
Metered‐dose inhaler						Group; *F* = 136.960; *p* < **0.001** Time; *F* = 828.872; *p* < **0.001** Group*Time; *F* = 781.382; *p* < **0.001**
Pretest		6.42 ± 2.14	7.29 ± 2.50	**0.013**	**−0.37 (−0.67; −0.08)**	
Posttest		15.23 ± 1.68	7.36 ± 2.51	**<0.001**	**3.65 (3.16; 4.14)**	
Test statistics^a^	*p*	**<0.001**	**0.023**			
	*d* (GA 95.0%)	**−3.01 (−3.52;‐2.50)**	**−0.24 (−0.45; −0.03)**			
Difference		8.84 ± 2.94	0.13 ± 0.54	**<0.001**	**4.23 (3.69; 4.77)**	
Dry‐powder inhaler‐1						Group; *F* = 18.450; *p* < **0.001** Time; *F* = 25.290; *p* < **0.001** Group*Time; *F* = 23.092; *p* < **0.001**
Pretest		8.61 ± 2.20	8.73 ± 1.19	0.874	−0.06 (−0.81; 0.69)	
Posttest		12.65 ± 0.70	8.82 ± 1.40	**<0.001**	**3.72 (2.45; 4.97)**	
Test statistics^a^	*p*	**<0.001**	0.588			
	*d* (GA 95.0%)	**−1.51 (−2.21;‐0.8)**	−0.17 (−0.76; 0.43)			
Difference		4.00 ± 2.65	0.09 ± 0.54	**<0.001**	**1.86 (0.94; 2.76)**	
Dry‐powder inhaler‐2						Group; *F* = 82.942; *p* < **0.001** Time *F* = 291.785; *p* < **0.001** Group*Time; *F* = 265.254; *p* < **0.001**
Pretest		7.83 ± 1.65	8.14 ± 1.13	0.203	−0.23 (−0.58; 0.12)	
Posttest		12.19 ± 1.15	8.19 ± 1.20	**<0.001**	**3.39 (2.83; 3.94)**	
Test statistics^a^	*p*	**<0.001**	0.34			
	*d* (GA 95.0%)	**−2.17 (−2.67; −1.67)**	−0.12 (−0.35; 0.12)			
Difference		4.40 ± 2.02	0.07 ± 0.63	**<0.001**	**3.06 (2.53; 3.59)**	
Nebulizer						Group; *F* = 4.326; *p* = 0.056 Time; *F* = 10.750; *p* = **0.005** Group*Time; *F* = 10.700; *p* = **0.006**
Pretest		5.89 ± 2.15	5.89 ± 1.05	0.999	0 (−0.92; 0.92)	
Posttest		8.67 ± 0.50	6.14 ± 1.07	**<0.001**	**3.17 (1.62; 4.68)**	
Test statistics^a^	*p*	**0.006**	0.999			
	*d* (GA 95.0%)	**−1.25 (−2.12; −0.34)**	0 (−0.92; 0.92)			
Difference		2.78 ± 2.22	0.00 ± 0.00	**0.006**	**1.65 (0.47; 2.79)**	

^a^ Within‐group comparison (Paired *t*‐test).

^b^ Between‐group comparison (Student's *t*‐test), effect size (*d*), mean (*X*), standard deviation (*SD*), confidence interval (CI), *F* (Greenhouse–Geisser test statistic), dof, degrees of freedom.

The findings obtained with ITT (*n* = 200) analysis and per‐protocol analysis, which were conducted to prevent bias in the research and to determine whether the participants who left the intervention group affected the research, are similar (Table 4).

**TABLE 4:** Comparison of pretest and posttest scores of intervention and control groups according to ITT analysis.

Variables		Intervention	Control	Test statistics^b^	
		*X* ± *SD*	*X* ± *SD*	*p*	*d* (GA 95.0%)
MARS					
Pretest		18.19 ± 5.00	19.00 ± 4.82	0.245	−0.17 (−0.44; 011)
Posttest		19.28 ± 4.63	18.43 ± 4.47	0.188	0.19 (−0.09; 0.46)
Test statistics^a^	*p*	**0.002**	**0.009**		
	*d* (GA 95.0%)	**−0.32 (−0.52; −0.12)**	**0.05 (0.07; 0.47)**		
Difference		1.09 ± 3.43	−0.57 ± 2.14	**<0.001**	**0.58 (0.30; 0.86)**
CAFS					
Pretest		61.25 ± 18.22	60.83 ± 17.49	0.869	0.02 (−0.25; 0.30)
Posttest		47.86 ± 14.85	62.78 ± 16.66	**<0.001**	**−0.95 (−1.24;‐0.65)**
Test statistics^a^	*p*	**<0.001**	0.073		
	*d* (GA 95.0%)	**0.86 (0.63; 1.09)**	0.06 (−0.38; 0.02)		
Difference		−13.39 ± 15.55	1.95 ± 10.77	**<0.001**	**−1.15 (−1.44; −0.85)**
ACT					
Pretest		10.90 ± 3.59	9.88 ± 3.20	**0.035**	**0.30 (0.02; 0.58)**
Posttest		16.55 ± 4.03	9.88 ± 3.74	**<0.001**	**1.71 (1.39; 2.04)**
Test statistics^a^	*p*	**<0.001**	0.993		
	*d* (GA 95.0%)	**−1.50 (−1.79; −1.22)**	0.07 (−0.20; 0.20)		
Difference		5.65 ± 3.75	0.00 ± 2.51	**<0.001**	**1.77 (1.44; 2.09)**
Metered‐dose inhaler					
Pretest		6.42 ± 2.14	7.29 ± 2.50	**0.013**	**−0.37 (−0.67; −0.08)**
Posttest		14.58 ± 2.82	7.42 ± 2.56	**<0.001**	**2.66 (2.26; 3.06)**
Test statistics^a^	*p*	**<0.001**	**0.023**		
	*d* (GA 95.0%)	**−2.21 (−2.59; −1.82)**	**0.02 (−0.45; −0.03)**		
Difference		8.16 ± 3.69	0.13 ± 0.54	**<0.001**	**3.07 (2.64; 3.50)**
Dry‐powder inhaler‐1					
Pretest		8.61 ± 2.20	8.73 ± 1.19	0.874	−0.06 (−0.81; 0.69)
Posttest		12.39 ± 1.29	8.82 ± 1.40	**<0.001**	**2.68 (1.63; 3.70)**
Test statistics^a^	*p*	**<0.001**	0.588		
	*d* (GA 95.0%)	**−1.38 (−2.02; −0.72)**	0.12 (−0.76; 0.43)		
Difference		3.78 ± 2.73	0.09 ± 0.54	**<0.001**	**1.68 (0.80; 2.54)**
Dry‐powder inhaler‐2					
Pretest		7.92 ± 1.76	8.14 ± 1.13	0.378	−0.16 (−0.50; 0.19)
Posttest		11.86 ± 1.61	8.21 ± 1.21	**<0.001**	**2.59 (2.12; 3.06)**
Test statistics^a^	*p*	**<0.001**	0.340		
	*d* (GA 95.0%)	**−1.69 (−2.09; −1.29)**	0.06 (−0.35; 0.12)		
Difference		3.95 ± 2.34	0.07 ± 0.62	**<0.001**	**2.36 (1.90; 2.81)**
Nebulizer					
Pretest		5.89 ± 2.15	5.89 ± 1.05	0.999	0.00 (−0.92; 0.92)
Posttest		8.67 ± 0.50	5.89 ± 1.05	**<0.001**	**3.37 (1.87; 4.83)**
Test statistics^a^	*p*	**0.006**	0.999		
	*d* (GA 95.0%)	**−1.25 (−2.12; −0.34)**	0.00 (−0.92; 0.92)		
Difference		2.78 ± 2.22	0.00 ± 0.00	**0.002**	**1.77 (0.64; 2.85)**

^a^ Within‐group comparison (Paired *t*‐test).

^b^ Between‐group comparison (Student's *t*‐test), effect size (*d*), mean (*X*), standard deviation (*SD*), confidence interval (CI).

Linear regression analysis was conducted to determine the effect of web‐based education on various outcome measures. The analysis results for the MARS variable (*β* = 0.010, *p* = 0.188, *R* ^2^ = 0.009) were not statistically significant, indicating low explanatory power of the model. For the CAFS variable (*β* = −0.012, *p* < 0.001, *R* ^2^ = 0.193), the results were statistically significant, showing a moderate level of explanatory power. The analysis for the ACT variable (*β* = 0.064, *p* < 0.001, *R* ^2^ = 0.447) indicated that web‐based education had a significant effect, with high explanatory power. The variables metered‐dose inhaler (*β* = 0.098, *p* < 0.001, *R* ^2^ = 0.771), dry‐powder inhaler‐1 (*β* = 0.204, *p* < 0.001, *R* ^2^ = 0.781), dry‐powder inhaler‐2 (*β* = 0.186, *p* < 0.001, *R* ^2^ = 0.741) and nebulizer (*β* = 0.293, *p* < 0.001, *R* ^2^ = 0.739) also showed significant effects of web‐based education with high explanatory values (Table 5).

**TABLE 5:** Linear regression analysis results to determine the effect of web‐based education on the variables.

Independent variable	*β*	*Beta*	*t*	*R*	*R* ^2^	Adjusted *R* ^2^	*F*	*p*	Durbin–Watson
MARS	0.010	0.96	1.322	0.096	0.009	0.004	1.747	0.188	0.033
CAFS	−.012	−.439	−6.725	0.439	0193	0.189	45.226	**0.000**	0.317
ACT	0.064	0.668	12.351	0.668	0.447	0.444	152.555	**0.000**	0.767
Metered‐dose inhaler	0.098	0.878	24.118	0.878	0.771	0.769	581.691	**0.000**	1.867
Dry‐powder inhaler‐1	0.204	0.884	9.618	0.884	0.781	0.772	92.506	**0.000**	1.467
Dry‐powder inhaler‐2	0.186	0.861	18.630	0.861	0.741	0.739	347.081	**0.000**	1.602
Nebulizer	0.293	0.860	6.296	0.860	0.739	0.720	39.643	**0.000**	2.505

Abbreviation: β, standardized regression coefficient.

When the site usage and compliance rates of the participants in the intervention group were analysed according to the weeks, it was found that the usage rate was 19.6% in the first week, 32.6% in the second week, 20.7% in the third week, 22.8% in the fourth week, 15.2% in the fifth week and 60.9% in the sixth week. In order to use the website, 92.4% of the participants stated that they did not need any help, whereas 6.5% stated that they needed help (Table 6).

**TABLE 6:** Distribution of website usage and compliance.

Weeks of use of the website	*n*	%
First week use	18	19.6
Second week use	30	32.6
Third week of use	19	20.7
Fourth week of use	21	22.8
Fifth week of use	14	15.2
Sixth week of use	56	60.9
**Needing help**	***n***	**%**
Yes	6	6.5
Partially	1	1.1
No.	85	92.4

## 5 DISCUSSION

The results of the present study, in a parallel‐group, randomized controlled experimental trial showed that the web‐designed education developed for asthma patients could asthma control, fatigue levels and inhalation devices usage techniques knowledge scores of individuals with asthma. However, it was found that this program did not improve drug adherence.

### 5.1 Findings on asthma control and fatigue levels

Achieving adequate asthma control means a reduction in asthma symptoms and exacerbations, reduced use of rescue medication, fewer nighttime awakenings and improved quality of life (Lozier et al., 2018; Sundberg et al., 2009). The results obtained in the present study showed that posttest CAFS scores decreased significantly in the intervention group compared with the control group, and posttest ACT scores were high in the intervention group. In addition, group*time had a significant effect on of CAFS and ACT. These results show that the intervention had a positive effect on the fatigue levels and asthma control of the patients in the intervention group. In several studies, internet‐based education resulted in a positive and statistically significant change in asthma control in the intervention group (Beerthuizen et al., 2020; Beerthuizen et al., 2021). In some studies, internet‐based self‐management support has been found to improve quality of life, number of symptom‐free days and clinical outcomes in patients with partially controlled asthma (Van der Meer et al., 2009; Van Gaalen et al., 2013).

### 5.2 Findings on drug adherence

Non‐adherence with asthma treatment is a significant burden for patients and communities. Non‐adherence encompasses poor initiation, execution (including poor inhalation technique) and persistence (Jansen et al., 2021). The results of the present study showed that posttest MARS scores increased significantly in the intervention group compared with the pretest scores, whereas a decrease was observed in the control group. Similarly, Wiecha et al. found a significant improvement in medication adherence in the intervention group (Wiecha et al., 2015). However, in several web‐designed studies, it was reported that there was no significant difference between the intervention and control groups in terms of medication adherence (Gustafson et al., 2012; Kolmodin MacDonell et al., 2016; Koufopoulos et al., 2016). The increase in medication adherence was most likely due to the participants in the intervention group accessing the website whenever they needed, reading the content and watching the videos related to the information they sought as much as and whenever they wanted.

### 5.3 Findings on inhalation devices usage techniques knowledge

Inhaled drug delivery is the cornerstone of the treatment of obstructive chronic airway diseases such as asthma (Gregory et al., 2013). The most common drugs used are pressurized metered‐dose inhalers and dry‐powder inhalers (Pritchard, 2015). Recent advances in inhaler technologies have led to a wide variety of device options (Lavorini et al., 2014). However, this creates confusion among healthcare providers and patients about their operation (Haughney et al., 2010). Several studies have shown that despite advances in inhaler device technology, technical errors made by patients with asthma are common (Lavorini & Usmani, 2013; Molimard et al., 2003; Molimard et al., 2017). Correct use of the inhaler is of primary importance in the treatment of asthma. There is a difference between not using an inhaler and using it incorrectly. The former can be intentional or unintentional; the latter is usually unintentional. That is, patients inhale their medication but not correctly (Sulaiman et al., 2017). The results obtained in the present study showed that metered‐dose inhaler, dry‐powder inhaler‐1, dry‐powder inhaler‐2 and nebulizer posttest scores increased significantly in the intervention group than those in the control group, whereas the pretest and posttest scores were similar in the control group. In addition, the group*time effect on metered‐dose inhaler, dry‐powder inhaler‐1 and dry‐powder inhaler‐2 scores was significant in measures. These results show that the web‐based education had a positive effect on the knowledge levels of the intervention group about inhaler use. In another educational study on inhaler technique conducted by Park et al. (2018), it was observed that the correct inhaler use increased in the intervention group.

### 5.4 Strengths and limitations

This study presents a number of strengths and limitations. Asthma is diagnosed based on anamnesis alone, excluding laboratory evaluations. Therefore, many patients are diagnosed with asthma even though they do not have asthma. This led to difficulties in identifying the people who met the inclusion criteria of the study.

A web‐designed approach helps to reach patients who do not want to participate in a traditional asthma education program and is important in terms of filling the gaps in the follow‐up of individuals with asthma and evaluating asthma control and medication adherence. However, web‐based approaches are more attractive to younger age groups, and the applicability of the method developed in the present study to people with limited health literacy and older age groups is a limitation of the study.

Furthermore, a limitation of this study is the lack of data on the ‘dose’ of the intervention, specifically the amount of time each participant spent engaged in the web‐based asthma education program. Future research should aim to collect and account for these data to better understand the relationship between engagement time and outcomes.

## 6 CONCLUSİON

This study demonstrates that the web‐designed education program developed for individuals with asthma significantly improves inhaler technique, positively impacts patients' fatigue levels and enhances asthma control. The program's effectiveness in teaching correct inhaler use and managing asthma symptoms underscores the potential of web‐based interventions in asthma care. However, although the program shows promise, its applicability may be limited for older adults and those with limited health literacy. Future research should focus on refining these educational tools to accommodate diverse populations, ensuring broader accessibility and efficacy. Additionally, integrating laboratory evaluations could enhance the accuracy of asthma diagnoses, further improving patient outcomes.

## AUTHORSHIP STATEMENT

Eylül Gülnur Erdoğan and Özlem Örsal HD designed the study. Eylül Gülnur Erdoğan was responsible for the education and counselling programme. Eylül Gülnur Erdoğan was responsible for data collection. Eylül Gülnur Erdoğan and Özlem Örsal were responsible for data analysis. Eylül Gülnur Erdoğan and Özlem Örsal prepared the manuscript. All authors approved the final version for submission.

## CONFLICT OF INTEREST STATEMENT

The authors declare no conflict of interest.

## CLINICAL TRIAL REGISTRATION NUMBER AND NAME OF TRIAL REGISTER

This study was registered at clinicaltrials.gov (NCT04607681).

## Data Availability

The data that support the findings of this study are available on request from the corresponding author. The data are not publicly available due to privacy or ethical restrictions.
